# Supplementary material for: Scalable Screening and Treatment Response Monitoring for Perinatal Depression in Low- and Middle-Income Countries
Source: Int J Environ Res Public Health. 2021 Jun 22;18(13):6693. doi: 10.3390/ijerph18136693 (PMC8297354; doi:10.3390/ijerph18136693)
Supplement: Supplementary file 1 [file ijerph-18-06693-s001.zip › ijerph-1137947-supplementary.pdf]

Supplementary Table S1: Criterion validity of the PHQ-4

Cut-off value	Sensitivity	1 - Specificity
-1.0000	1.000	1.000
.5000	.989	.462
1.5000	.989	.325
2.5000	.989	.232
3.5000	.978	.162
4.5000	.934	.083
5.5000	.868	.060
6.5000	.769	.034
7.5000	.615	.023
8.5000	.495	.011
9.5000	.374	.006
10.5000	.220	.002
11.5000	.154	.000
13.0000	.000	.000

Supplementary table S2: Criterion validity of HAMD-4 post-intervention

Cut off value	Sensitivity	1 - Specificity
-1.0000	1.000	1.000
.5000	.961	.361
1.5000	.951	.306
2.5000	.951	.220
3.5000	.942	.122
4.5000	.912	.075
5.5000	.776	.039
6.5000	.545	.016
7.5000	.377	.010
8.5000	.279	.008
9.5000	.214	.004
10.5000	.133	.004
12.0000	.000	.000
